# Case Report: Trans-axillary Artery Access for Rescue Stent Implantation in an Infant With Retrograde Non-passable Aortic Coarctation

**DOI:** 10.3389/fped.2021.625011

**Published:** 2021-04-08

**Authors:** Julia Moosmann, Ariawan Purbojo, Susanne Eder, Sven Dittrich

**Affiliations:** ^1^Department of Pediatric Cardiology, Friedrich-Alexander University of Erlangen-Nürnberg (FAU), Erlangen, Germany; ^2^Department of Pediatric Cardiac Surgery, Friedrich-Alexander University of Erlangen-Nürnberg (FAU), Erlangen, Germany

**Keywords:** congenital heart defect, coarctation of aorta, axillary artery access, stent implantation, vascular access

## Abstract

Primary surgical repair remains the traditional treatment for patients with critical duct-dependent coarctation of the aorta (CoA). Initial surgical repair might not be possible or associated with higher risks if additional comorbidities arise in small infants and neonates. Balloon angioplasty (BA) has been described as a rescue strategy for these children. We describe the feasibility of a palliative BA and rescue stent implantation via an alternative antegrade right-axillary artery approach in an initially inoperable infant with pneumonia and respiratory failure and severe CoA, where the stenosis was not passable by traditional retrograde femoral access. This case adds new aspects to the therapy of critical CoA: Stent implantation provides a bridge to surgery in critically ill infants and does not preclude successful surgical repair. Further, if the classic retrograde approach is not possible, the right axillary artery access should be considered as an alternative to pass the stenosis.

## Introduction

Coarctation of the aorta (CoA) accounts for 5–8% of all congenital heart defects (CHD). Surgical repair remains the traditional treatment of critical duct-dependent CoA in newborns and small infants. However, choice of treatment depends on a number of factors including age, weight and clinical condition accounting for the overall risk of the surgical procedure. Balloon angioplasty (BA) and stent implantation is associated with less acute complications compared to surgery, but they are more likely to require planned reintervention (1, 2).

The axillary artery (AA) access has gained more interest as an alternative vascular access in children especially for duct stenting and CoA (3–5). However, it still represents an often-unused access route.

In this report, we present a case of a critically ill infant who underwent palliative stenting of severe CoA by AA access, where retrograde passage of the stenotic segment was not possible.

## Case Presentation

A 3-month old infant (4.6 kg) was admitted to an external hospital with Respiratory Syncytial Virus (RSV) bronchiolitis and secondary *Haemophilus influenzae* Type B pneumonia with respiratory failure needing invasive ventilation. Ventilation quickly became challenging, requiring escalation to high frequency oscillation (HFO) ventilation. The pulmonary situation did not improve and the child developed additional cardiac decompensation requiring high inotrope support and two short periods of cardio pulmonary resuscitation. The patient was transferred to our pediatric cardiac intensive care unit (ICU). On initial examination the patient presented in a significantly reduced clinical condition, with prolonged recapillarisation time of 5 s, skin mottling, cold peripheries and no palpable femoral pulses, an active precordium with a 3/6 systolic murmur. X-ray showed diffuse bi-pulmonal infiltrations, a left pleural effusion and atelectasis of the left upper lobe.

Transthoracic echocardiography revealed severe CoA with minimal antegrade blood flow and an associated systolic peak pressure gradient of 65 mmHg in the presence of a severely impaired left ventricular (LV) function (FS 23%). The arterial duct was almost closed. The LV was hypertrophic with a mild mitral valve regurgitation. The right ventricular function was also impaired and pulmonary artery hypertension present (Pulmonary acceleration time: 25 ms and right ventricular pressure >60 mmHg). Considering the clinical condition with severe biventricular congestive heart failure and respiratory failure, we opted against a primary surgical repair in favor of an emergency interventional transcatheter rescue strategy.

**Figure 1:**
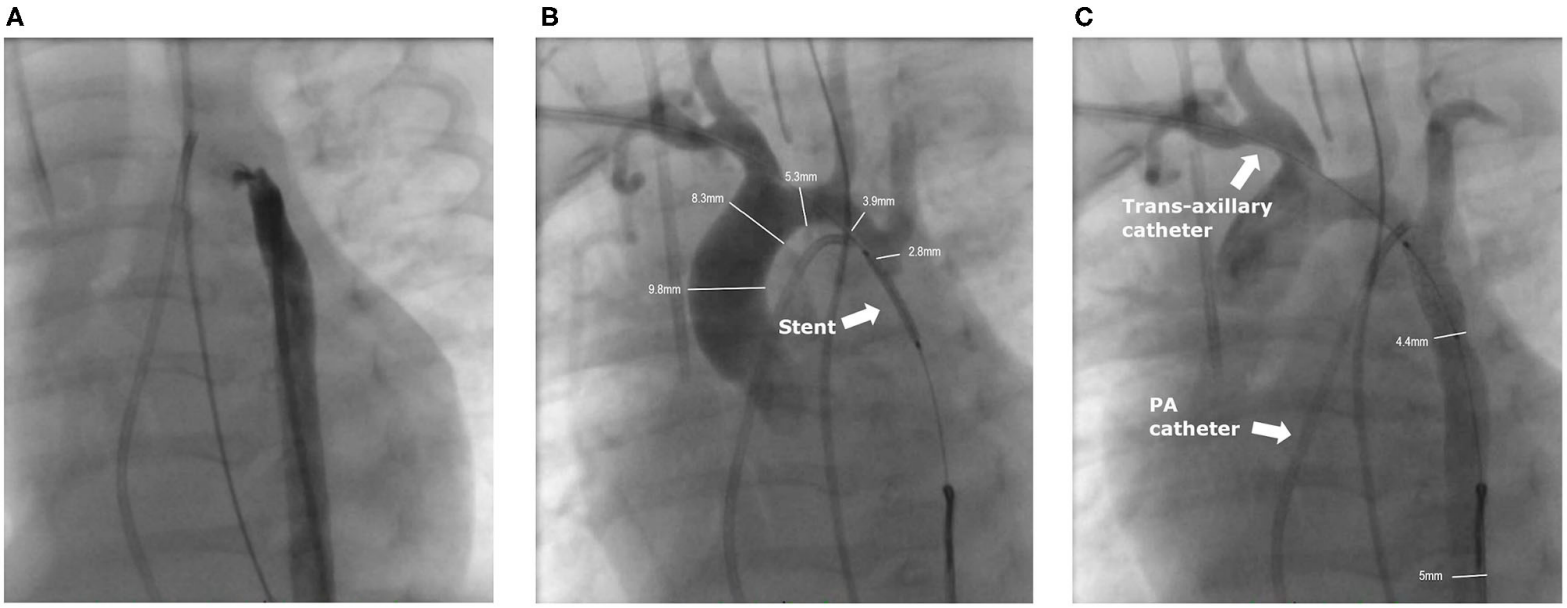
Angiographies of the intervention. **(A)** Angiography from the femoral artery. The small duct is slightly contrasted, but no contrast medium crosses the stenosis of the CoA. **(B)** The ironman wire through the right axillary sheath is snared in the descending aorta and the pre-mounted stent is in position (arrow) after pre-dilation of the CoA with a 2.5 mm coronary balloon. Contrast medium is applied through the 4F axillary sheath into the truncus brachiocephalicus. The non-inflated balloon completely obstructed the CoA. **(C)** Angiography after stent implantation with good position. A 4F multipurpose catheter is placed from the venous side in the small PDA (not contrasted).

The right femoral artery was first accessed for the intervention. However, a guide wire could not be advanced across the CoA due to the severity of the stenosis (Figure 1A). The wire passed through the slightly open arterial duct into the pulmonary artery (PA). Severe pulmonary hypertension was diagnosed with a peak PA systolic pressure of 106 mmHg and mean pressure of 79 mmHg.

To access the stenosis a 4F-sheath (Terumo; Somerset, New Jersey, US) was inserted ultrasound guided in the right AA. Antegrade passage of the nearly closed aortic isthmus was achieved with a 0.021-inch Teflon-coated wire of a 2.7 F Progreat microcatheter (Terumo; Somerset, New Jersey, US). The invasively measured pressure values in the transverse aortic arch proximal to the stenosis (114/40/71 mmHg) and in the descending aorta (DAO) distal to the stenosis (47/40/43 mmHg) corresponded to an invasively measured gradient of 67 mmHg, respectively.

The Progreat catheter was snared by a 5 mm Amplatzer Goose Neck Snare (Medtronic; Dublin, Ireland) from the DAO to build a stable loop. Via the Progreat microcatheter the wire was exchanged to a 0.014-inch Ironman wire (Abbot; Chicago, Illinois, US), and snared again in the DAO. The Progreat catheter was removed and a 2.5 × 20 mm Ryujin Plus balloon (Terumo; Somerset, New Jersey, US) was advanced and positioned over the CoA. After confirming the correct position, the stenosis was pre-dilated with 12.5 atm. After the pre-dilatation a pre-mounted 5.0 x 13 mm Pro kinetic Energy Stent (Biotronic, Berlin, Germany) was advanced. Angiography revealed extreme CoA and the pre-mounted stent completely occluded the lumen with angiographically no visible forward flow to the DAO (Figure 1B). The stent was expanded to 5.5 mm with a balloon pressure of 14 atm. The angiography showed the desired result with a harmonically modeled stent and a mild residual gradient of 17 mmHg (Figure 1C). After the intervention, the axillary sheath was removed and the AA carefully compressed. The patient received a single intravenous dose of 100 U heparin after placement of the femoral sheath, followed by a continuous heparin infusion of 10 U/kg/h.

**Figure 2:**
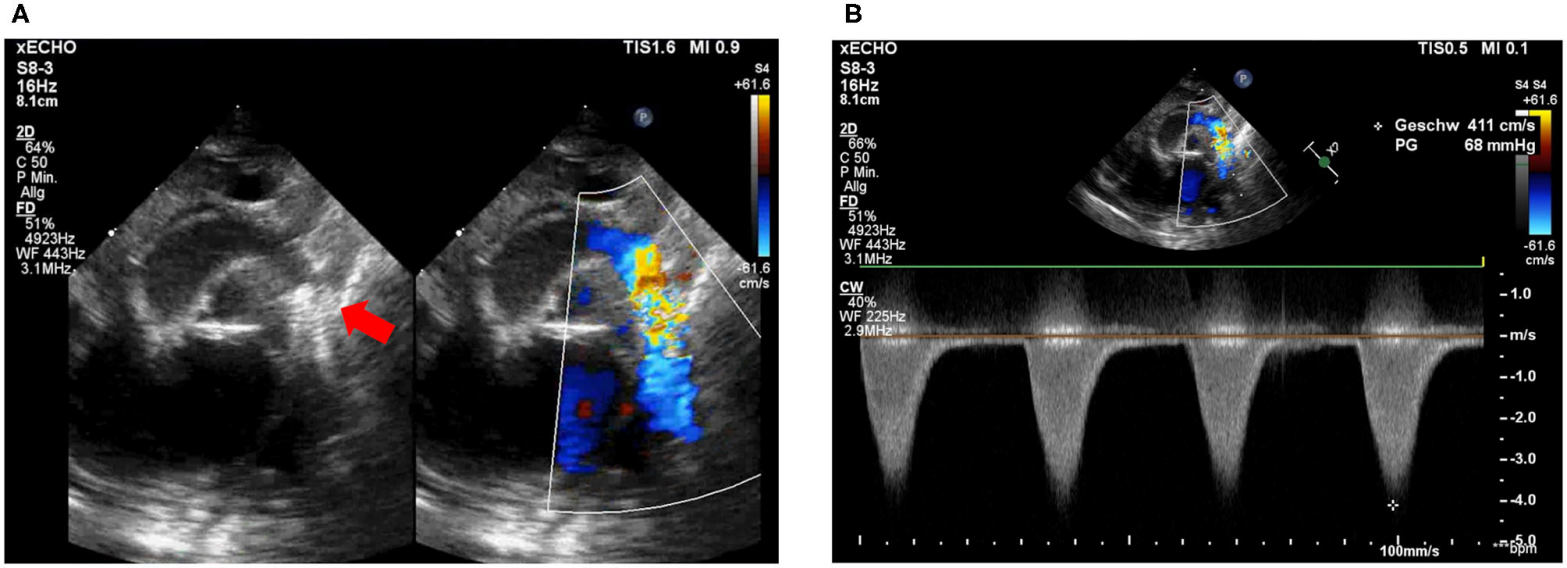
Post-intervention echocardiography. **(A)** Stent visualization in the descending aorta. Proximal stent struts (red arrow) partially cover the opening of the left subclavian artery (LSA) without obstruction. **(B)** Flow acceleration over coronary stent in isthmus region with 4.1 m/s.

**Figure 3:**
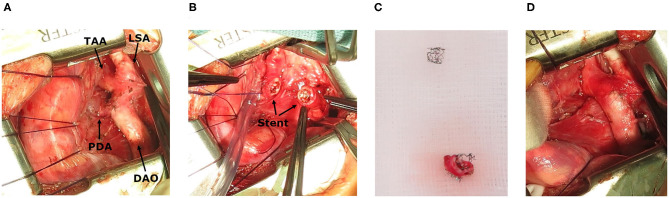
Intraoperative images. **(A)** Situs after stent implantation, showing transverse aortic arch (TAA), left subclavian artery (LSA) and descending aorta (DAO) and persistent ductus arteriosus (PDA). **(B)** Stent in the isthmus region. **(C)** Resection of coarctation with stent. **(D)** Surgical result.

After the procedure, the patient remained on the cardiac ICU and stabilized over the next days. Pneumonia and pulmonary edema improved and extubation was achieved 4 days after the intervention. Despite clinical improvement, echocardiography still showed a peak gradient over the stent of 64 mmHg (Figure 2) so that surgical therapy was still indicated. The patient underwent surgical stent removal and extended resection with end-to-end anastomosis and duct ligation one week after the intervention. The stent was easily removed during surgery (Figures 3A–D). The patient showed an uncomplicated postoperative course and was extubated a few hours after the surgery. She was discharged 2 weeks after the intervention in a good clinical condition with no residual non-invasive blood pressure gradient between right arm (82/53 mmHg) and leg (81/59 mmHg). Echocardiography at discharge showed a mildly accelerated flow of 2.1 m/s over the isthmus region, LV function was still impaired with a FS of 18% and LV hypertrophy. Due to the reduced LV function and arterial hypertension therapy with Enalapril was started.

Follow-up visits were performed 2 and 4 weeks after discharge showing no clinical signs of heart failure and a peak Doppler-gradient of 16 mmHg over the isthmus region. Six months and 1.5 years after the intervention, ventricular function (FS > 28%) and LV hypertrophy have normalized, the aortic arch shows a great surgical result and the patient presents in a good clinical condition.

## Discussion

We describe successful antegrade BA and stent implantation via the AA for severe CoA in a critically ill infant with LV heart failure, pulmonary artery congestion and respiratory failure as a bridge to surgical repair.

BA or stent implantation for duct-dependent native CoA has its limitations with a higher rate of reinterventions, aneurysm formation and especially in neonates trauma or thrombosis of the accessed artery (6, 7). Primary surgical therapy should therefore be preferred if possible. However, in critically ill newborns and infants with additional comorbidities an interventional approach has evolved as a life-saving treatment option, bridging to corrective surgery (8, 9). The interventional approach, especially with stent implantation, remains reserved for a selected group of patients and is mainly performed by conventional retrograde femoral access (8, 9). In our case, under emergency conditions, we were forced to choose an alternative access route because the stenosis was so severe that it could not be passed by a traditional retrograde approach. Schranz and Michel-Behnke described a detailed description of the AA access for cardiac interventions in newborns and promote it as an attractive alternative approach especially compared to carotid and femoral access (3). Other authors describe the AA access for BA of the aortic valve or CoA in neonates as a safe but underrepresented access (4, 5, 10). The AA access can be even easier in patients with CoA and LV failure presenting with decreased perfusion of the femoral artery. Therefore, AA remains easier to palpate and access, while femoral pulses and perfusion are weakened. One could argue that the carotid artery access offers the same advantages, which has been used successfully by others (11, 12). However, vascular complications including occlusion, thromboembolism or severe hematoma, which may occur especially in small children, are not as relevant using the AA compared to the carotid artery. Especially as it is not an end artery and arm perfusion remains guaranteed during the procedure. In our case, heparin was given after insertion of the sheath in the femoral artery as per our protocol. Therefore, we also decided to use the AA access, since puncture of the carotid artery under heparinization has higher risks.

AA access has been described previously for stent implantation for CoA e.g., in a case series of neonates with stable hemodynamics and preserved ventricular function (13). In contrast to our patient, the inventions were electively planned with a surgical exposure of the AA punctured under direct vision. This approach requires surgical repair of the AA at the end of the procedure. Esmaeili et al. recently pushed boundaries using the AA access for a planned stent implantation in a neonate with 1.2 kg, severe CoA and pulmonary hypertension (14).

CoA-stenting as a rescue maneuver is less invasive compared to surgical therapy. This allows the patient additional time to recover clinically as well as from LV dysfunction before undergoing surgical repair. Concerns that the stent may complicate surgical therapy could not been confirmed in our case, as it was easy to removed after 1 week (Figures 3A–D) (8, 15).

Despite our case clearly describes a severe congenital CoA complicated by pulmonary infection, we would like to point out the interesting correlation between *Haemophilus influenzae* infections and aortic aneurysm or aortitis, which have been reported previously (16, 17).

This report is limited to a single case, however adds new aspects to the therapy of critically ill children with severe CoA. Firstly, rescue stent implantation for patients with severe compromised hemodynamics provides a successful bridge to surgery. Secondly, the right AA access is an attractive alternative for patients with severe narrowing of the isthmus where traditional retrograde passage cannot be achieved. The percutaneous access of the AA can safely be performed ultrasound guided by an experienced interventionist and does not require surgical cut down.

## Conclusion

Percoutaneous AA access for antegrade BA and stenting represents an attractive alternative approach in critically ill infants with severe CoA who require emergency intervention and conventional retrograde passage is not possible. Rescue stent implantation does not preclude successful surgical repair if removal of the stent follows timely.

## Data Availability Statement

The raw data supporting the conclusions of this article will be made available by the authors, without undue reservation.

## Ethics Statement

Ethical review and approval was not required for the study on human participants in accordance with the local legislation and institutional requirements. Written informed consent to participate in this study was provided by the participants' legal guardian/next of kin.

## Author Contributions

SD performed the intervention. JM, SE, and SD were involved in the clinical decision making and care plan of the child. AP performed the surgery. JM and SD collected the clinical and scientific findings and wrote the manuscript. All authors discussed the results and contributed to the final manuscript.

## Conflict of Interest

The authors declare that the research was conducted in the absence of any commercial or financial relationships that could be construed as a potential conflict of interest.
